# Editorial: From structure to agency: understanding nurse's agency in quality and safe care

**DOI:** 10.3389/fpsyg.2023.1223807

**Published:** 2023-08-01

**Authors:** Einav Srulovici, Anat Drach-Zahavy

**Affiliations:** The Cheryl Spencer Department of Nursing, The University of Haifa, Haifa, Israel

**Keywords:** quality of care, safety, nurses, accountability, system-level, personal

Approximately 24 years have passed since the publication of the Institutes of Medicine (IOM) influential report “to err is human” (1999). In the report, devastating statistics were presented for the first time, regarding patient deaths due to preventable events, asserting that most deaths result from systemic errors rather than professional negligence. According to the authors, organizational changes should be implemented in order to improve quality and safety of healthcare services (Kohn et al., 1999). The publication of this highly significant report sparked the emergence of three waves of efforts to address the quality-of-care challenge within the healthcare system (See Figure 1). In the first wave, until the publication of the report, the focus was on the professional's accountability. Nursing scholars and policy makers generally believed that nurses' lack of motivation and/or knowledge contributed to poor quality and safety of care. Thus, organizations implemented a poor toolkit designed to improve quality of care, including standardization through procedures and protocols, training, and sanctions against those who failed to comply. However, research has concluded that this approach does not achieve its goals, since the main challenge is not preventing “bad” nurses from making mistakes, but preventing “good” nurses from making errors (Wynia and Classen, 2011; Edmondson, 2012).

**Figure 1:**
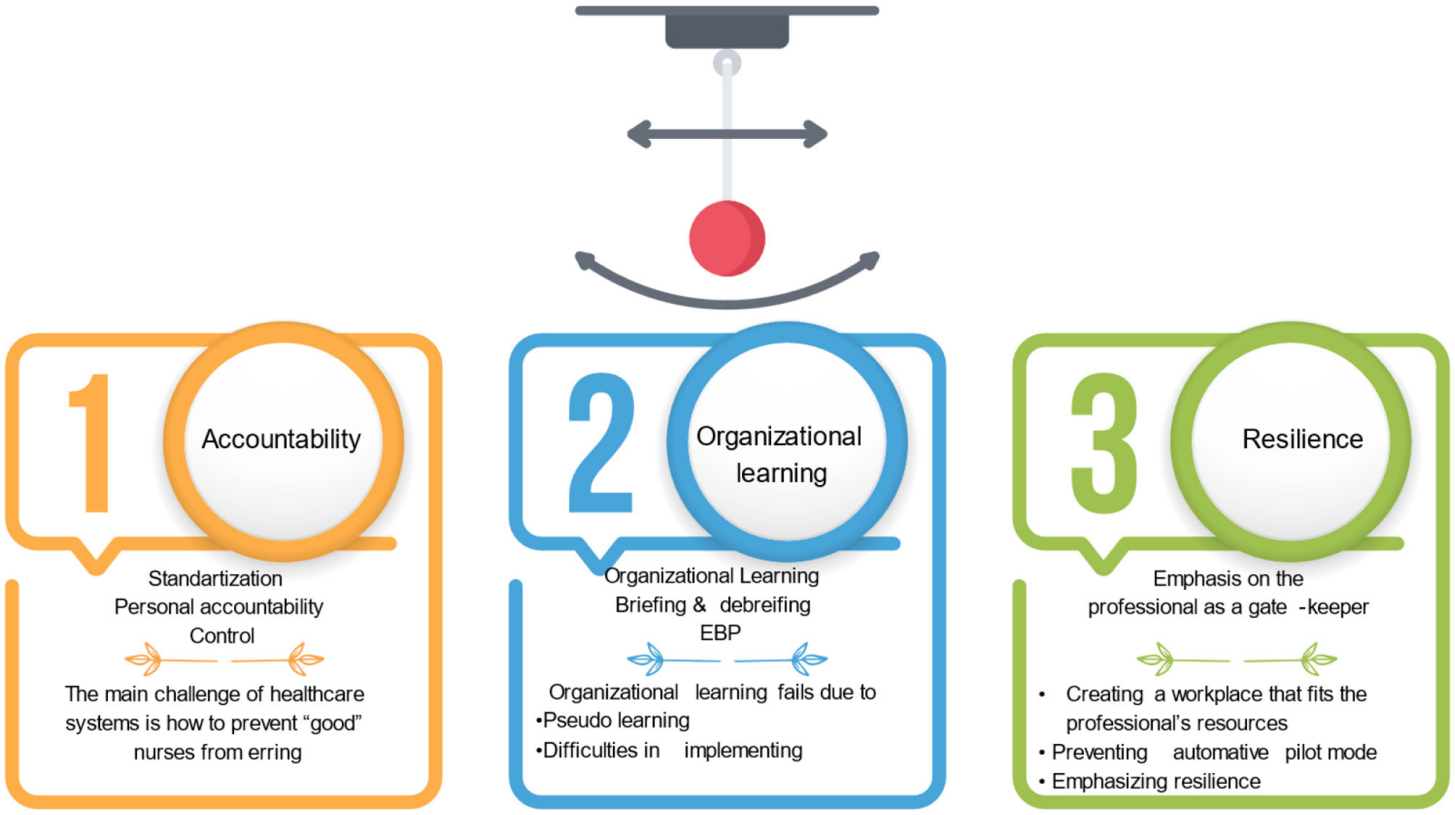
Three waves of the safety movement in healthcare settings.

In the second wave of efforts aimed at improving healthcare organizations' safety and quality, organizational learning has been emphasized. It included encouraging professionals to report errors and near-misses, implementing evidence-based nursing, and drawing conclusions regarding how to prevent similar mistakes in the future by participating in joint learning, inquiries, and risk management activities (Drach-Zahavy et al., 2014). However, examining the hidden agenda behind these actions to improve quality of care leads to the conclusion that the basic assumptions remained unchanged: nurses do not know how to provide quality of care, so we should encourage them to follow evidence-based nursing practices; or nurses are not motivated to improve the quality of care- so we should involve them in the learning process, as a way to improve their motivation. This wave of organizational learning did not produce the desired results, primarily because the learning was not significant, but rather seemed “pseudo-learning”, which makes it difficult for the conclusions to be implemented effectively (Edwards, 2017; Guttman et al., 2021). Additionally, understanding why an error occurred does not necessarily lead to its prevention in the future. Thus, scholars regrettably agree that despite the merit of looking at system problems, and highlighting organizational learning, the healthcare system has not shown enough improvement (Bates and Singh, 2018). Authors suggest that perhaps, the pendulum swung too far toward systems, thus should now swing back toward individual agency (Wachter and Pronovost, 2009; Latney, 2016). It is believed that most hospital wards and primary care clinics are now facing this second wave of striving for better quality of care.

The third wave, which emphasizes the importance of personal and organizational resilience, suggests that we might have overshot our mark with the notion that to err is human, and advocates a greater balance between individual accountability and systemic explanations (Wachter and Pronovost, 2009). Researchers show a renewed interest in nurses' agency, including nurses” competence, personal traits and values, and decision-making processes, raising questions about designing work environments that will enable nurses to thrive and deliver high quality care despite the complex, overburdened, and dynamic environments in which they operate (Drach-Zahavy and Srulovici, 2019; Abdelhadi et al., 2023). It emphasizes developing a culture that fosters a critical mind-set, a commitment to early detection of negligence and unexpected events, as well as building behavioral capabilities for proactive behaviors that assure rapid adjustment, and prevent patients' circumstances from worsening (Hales and Chakravorty, 2016; Latney, 2016; Vogus and Singer, 2016; Enya et al., 2018).

Our Research Topic focuses on this third wave. It sought to focus attention on nurses' work structures and their agency in affecting patient safety and quality of care. Two studies examined nurses' agency and capabilities to enhance quality of care. In Sperling et al. study, nurse champions were viewed as street-level bureaucrats. The authors looked at factors that support nurses' abilities to generate radical change at the grassroots level in their workplace, rather than looking at them as passively responding to constraints imposed by systems. According to the authors, nurse champions who ask their colleagues within the field for advice are more likely to implement radical changes. The establishment, support, and promotion of heterogeneous, dense professional networks are necessary to support advice sharing. Hu et al. also found in their study that nurses' proactive personality promoted performance by impacting nurses' engagement and competence. These two studies describe how nurses' agency can promote quality of care.

Sharon et al. examined nurses' agency from a different perspective, by systematically reviewing and meta-analyzing the literature related to motivating employees' performance in the workplace, whether through outcome- or process-based accountability. In the study, outcome accountability improved performance for more-complex tasks, whereas process accountability improved performance for simpler tasks. These findings are essential for nursing, which places a greater emphasis on outcomes than on processes, such as in the case of national quality indicators. In this study, the results illustrate how nurses' motivation and performance can be increased by tailoring motivation mechanisms to nurses' workplace circumstances, thus demonstrating the importance of balancing system and nurse concerns.

Finally, two studies investigated how to lead nurses to promote quality of care. Marques-Quinteiro et al. studied authentic leadership as a job resource that facilitates nurses' performance. According to the results, overload might serve as an organizational constraint that limits the effectiveness of authentic leadership. According to Witczak et al., safety culture has a negative relationship with missed nursing care. Based on the findings, missed nursing care should not be viewed as a “necessary evil” that cannot be avoided because of limited resources. Rather, by cultivating a safety culture, we can limit the devastating effects of this phenomenon. Together, the papers provide preliminary guidelines for developing an overarching agency-sensitive theory that examines how to manage the balance across multiple levels of analysis.

## Author contributions

All authors listed have made a substantial, direct, and intellectual contribution to the work and approved it for publication.
